# Sex and Genotype Influence Neural Progenitor Cells in a Sporadic Alzheimer's Disease Model

**DOI:** 10.1002/alz70855_102174

**Published:** 2025-12-23

**Authors:** Xinyi Lin, Samantha A Blankers, Kimberly Go, Bonnie H Lee, Liisa AM Galea

**Affiliations:** ^1^ University of Toronto, TORONTO, ON, Canada; ^2^ Centre for Addiction and Mental Health, TORONTO, ON, Canada; ^3^ Centre for Addiction and Mental Health, Toronto, ON, Canada; ^4^ University of British Columbia, Vancouver, BC, Canada; ^5^ Centre for Addiction and Mental Health, Campbell Family Mental Health Research Institute, Toronto, ON, Canada

## Abstract

**Background:**

The three non‐modifiable risk factors for Alzheimer's disease (AD) are advancing age, female sex and at least one APOEε4 allele. Females with AD experience more severe cognitive decline and pathological changes in the brain, including faster hippocampal atrophy, greater AD neuropathology, and steeper cognitive decline than males with AD. Notably, female APOEε4 carriers have an even higher risk of developing AD earlier, exhibiting greater cognitive decline and more severe neuropathological outcomes than male APOEε4 carriers at middle age. Hippocampal neurogenesis is altered in AD, and sex differences have been identified. However, the effect of advancing age, sex, and APOE genotype on neurogenesis remains unexplored. Therefore, the current study aims to identify how sex and APOE genotype differently affect the dynamics of neurogenesis, including the expression of neural progenitor cells and the maturation rate of new neurons.

**Method:**

We used 2‐month‐old (young adulthood) and 12‐month‐old (middle age) male and female humanized (h) APOEε3 and hAPOEε4 mice. Newly proliferated cells were labeled with the thymidine analog bromodeoxyuridine (BrdU). Brains were collected either 24 hours, 2 weeks, or 4 weeks after BrdU injection to capture the various stages of neurogenesis. The extracted brains were sectioned and processed for immunohistochemistry to measure the neural progenitor cell pool (BrdU/Sox2), the maturation of new neurons (BrdU/NeuN), and potentially the pluripotency of newly formed putative neural stem cells (BrdU/Sox2‐ir).

**Result:**

Analyses are ongoing, but we expect that hAPOEε4 mice will display different temporal dynamics of neurogenesis compared to hAPOEε3 mice, which will result in different levels of hippocampal neurogenesis. Moreover, interactions between sex and genotype are anticipated, with hAPOEε4 females expected to exhibit a slower neuronal maturation rate compared to other groups.

**Conclusion:**

Our research findings will reveal the role of sex and genotype in neurogenesis, neuronal maturation and factors influencing the pluripotency of neural stem cells in AD model, providing a better understanding of AD‐related neuropathological changes.

